# Environmental Heterogeneity Explains the Genetic Structure of Continental and Mediterranean Populations of *Fraxinus angustifolia* Vahl

**DOI:** 10.1371/journal.pone.0042764

**Published:** 2012-08-08

**Authors:** Martina Temunović, Jozo Franjić, Zlatko Satovic, Marin Grgurev, Nathalie Frascaria-Lacoste, Juan F. Fernández-Manjarrés

**Affiliations:** 1 Department of Forest Genetics, Dendrology and Botany, Faculty of Forestry, University of Zagreb, Zagreb, Croatia; 2 Department for Seed Science and Technology, Faculty of Agriculture, University of Zagreb, Zagreb, Croatia; 3 State Institute for Nature Protection, Zagreb, Croatia; 4 AgroParisTech, Laboratoire Ecologie, Systématique et Evolution, UMR 8079, Orsay, France; 5 Univ. Paris-Sud, UMR 8079, Orsay, France; 6 CNRS, UMR 8079, Orsay, France; University of Lausanne, Switzerland

## Abstract

Tree species with wide distributions often exhibit different levels of genetic structuring correlated to their environment. However, understanding how environmental heterogeneity influences genetic variation is difficult because the effects of gene flow, drift and selection are confounded. We investigated the genetic variation and its ecological correlates in a wind-pollinated Mediterranean tree species, *Fraxinus angustifolia* Vahl, within a recognised glacial refugium in Croatia. We sampled 11 populations from environmentally divergent habitats within the Continental and Mediterranean biogeographical regions. We combined genetic data analyses based on nuclear microsatellite loci, multivariate statistics on environmental data and ecological niche modelling (ENM). We identified a geographic structure with a high genetic diversity and low differentiation in the Continental region, which contrasted with the significantly lower genetic diversity and higher population divergence in the Mediterranean region. The positive and significant correlation between environmental and genetic distances after controlling for geographic distance suggests an important influence of ecological divergence of the sites in shaping genetic variation. The ENM provided support for niche differentiation between the populations from the Continental and Mediterranean regions, suggesting that contemporary populations may represent two divergent ecotypes. Ecotype differentiation was also supported by multivariate environmental and genetic distance analyses. Our results suggest that despite extensive gene flow in continental areas, long-term stability of heterogeneous environments have likely promoted genetic divergence of ashes in this region and can explain the present-day genetic variation patterns of these ancient populations.

## Introduction

Understanding how environmental heterogeneity influences the distribution of genetic variation among natural populations along different spatial scales remains a central question in evolutionary biology and population genetics [1], [2]. Genetic divergence of natural plant populations can be influenced by several evolutionary processes including gene flow, genetic drift, and natural selection [3]. If gene flow is locally restricted because of limited pollen and seed dispersal of the species, then the genetic differentiation of populations will show a pattern of isolation-by-distance (IBD) [4], which is considered to be the main force in the establishment of neutral genetic structure in plant populations.

Yet, greater genetic divergence than expected among populations inhabiting different environments has been used to suggest that contrasting ecological conditions may have a strong influence on the genetic differentiation of local populations [5], [6]. Several studies have shown statistical associations between putatively neutral genetic variation and environmental variation in plant species, and such correlations may be interpreted as evidence of diversifying selection acting over the whole genome [3], [7]–[9]. In cases in which the genetic distance between populations correlates with their environmental distance, the pattern has been described as “isolation by environmental distance” (IBED) [10]. Nevertheless, the removal of geographic effects is necessary to detect the unique contribution of environmental gradients to genetic divergence because climatic differences are typically correlated with geographic distance [11]. A significant positive partial correlation after removing the effect of geographic distance suggests that genetic divergence is associated with environmental gradients and that natural selection may interact with neutral processes of gene flow and genetic drift [9]–[11].

Ecological niche modelling (ENM) allows the generation of biogeographical hypotheses and, when coupled with genetic data, provides new insights into the evolutionary history of animal and plant species [11]–[15]. For instance, ENM indicates that two population sets of unresolved taxonomic status with non-overlapping ecological niches and separated by a portion of unsuitable habitat may represent distinct evolutionary lineages ([16], [17], see example for geckos in Madagascar in [12]). As such, the application of ENM to delimit cryptic species has become an emergent field of ecological genetics [11], [17], [18]. In particular, the ability of ENM to test for a potential lack of spatial overlap in subpopulations of the same species allows the generation of different gene flow hypotheses that otherwise would be difficult to formulate. For example, if population differentiation with neutral markers is low but ENM simulations produce non-overlapping distributions, one can conclude that gene flow is still present despite the contrasting habitats associated with the populations. On the other hand, if genetic structuring of neutral markers reflects the simulated spatial distributions, rates of gene flow must be interrupted to allow for population differentiation. Although linking ENM with genetic data is increasing rapidly in phylogeographic studies at broad scales, the potential of the ENM approach and GIS-based environmental data has rarely been explored in conjunction with population genetic variation at finer scales. Studies of landscape genetics [1], [19], which investigate how environmental factors influence the spatial distribution of intraspecific genetic variation, provide a promising framework for a better understanding of the microevolutionary processes involved in population genetic differentiation. While the majority of landscape genetics studies remain focused on animals [20], the integration of environmental features to explain the genetic variation of plants lags behind.

For this study, we selected the narrow-leaved ash (*Fraxinus angustifolia* Vahl, Oleaceae), a wind-pollinated tree species with a predominantly Mediterranean distribution, but found frequently in habitats of both Continental and Mediterranean biogeographical regions of Europe. It occurs naturally throughout Southern and Eastern Europe, from Portugal in the west to the Black Sea in the east. Although the phylogeography and genetic structure of the closely related species, the common ash (*Fraxinus excelsior* L.), and the hybridisation process between the two species have been well studied [21]–[26], no attempts have been made to understand the genetic structure of *F. angustifolia* populations. Unlike *F. excelsior*, which is widely distributed in mixed deciduous forests all over Europe, *F. angustifolia* is a habitat specialist associated with surface and ground waters, and thus, its dispersal ability is more restricted. In Central Europe, the Pannonian Basin and the Balkans, it occurs mainly in lowlands, riparian and floodplain forests along large rivers and their tributaries (Drava, Danube, and Morava), where it forms large and continuous populations. The distribution of this species in the Mediterranean region is patchy and reduced to smaller and more isolated populations on drier sites at higher altitudes or on wetland sites [27], [28].

There are several different points of view regarding the taxonomic status of this species, but in general, the prevailing opinion is that *F. angustifolia* has three subspecies restricted by geographical regions [27], [29]: ssp. *angustifolia* (in the western Mediterranean), ssp. *oxycarpa* (M. Bieb. ex Willd.) Franco and Rocha Afonso (in East Central and Southeastern Europe), and ssp. *syriaca* (Boiss.) Yalt. (in Turkey and eastwards to Iran). Fukarek [30] used morphological differences to further subdivide *F. angustifolia* in Croatia and the surrounding area of the Western Balkans. Continental populations along the rivers and floodplains of the Danube River Basin were named *Fraxinus angustifolia* ssp. *pannonica* (Fuk.) Soó and Simon. This putative new taxon is morphologically closer to ssp. *oxycarpa*, whereas Mediterranean populations along rivers and wetlands of the Adriatic River Basin probably belong to the typical ssp. *angustifolia*. Such a division differs from the more accepted general geographical classification, suggesting a possible increased within-region differentiation in the Western Balkan area.

Although located at a relatively high latitude and with a relatively small size, Croatia has been highlighted as having one of the most genetically divergent forests among 25 European forests investigated based on chloroplast genetic diversity [31]. Moreover, the area of the Western Balkans and Dinaric Alps served as an important refugium during the Pleistocene for the survival of many animal and plant species, including ash [23], [32]–[34]. Such large genetic divergence may have originated because of the comparatively higher environmental stability of this area during Quaternary climate oscillations, complex historical demographic events, and its geographical position. In fact, Croatia is situated along the contact line of three different biogeographical regions of Europe with contrasting climates: the Continental region including parts of the Pannonian lowlands, the mountainous Alpine region including parts of the Dinaric Alps and the Mediterranean region. Such environmental, landscape and historical diversity in a small area represents a valuable opportunity to investigate the influence of these factors on genetic variation and possible differentiation within species lineages.

In this study, we combined population genetic analyses of neutral loci, landscape genetic analysis using multivariate environmental data and ENM to examine genetic variation and its ecological correlates in *F. angustifolia* populations. We focused on answering these specific questions: 1) What are the levels of genetic diversity and divergence within and among natural populations of *Fraxinus angustifolia* distributed across the Croatian Continental and Mediterranean areas? 2) Does the neutral spatial genetic variation correlate with the environmental variation? and 3) Are areas predicted as suitable by ENM concordant with patterns of population genetic divergence?

## Materials and Methods

### Ethics statement

Collections of samples from protected areas were permitted by the authority of The Krka National Park and Lonjsko Polje Nature Park. For other locations no specific permits were required for the described field studies because sample collection did not involve endangered or protected plant species or privately-owned locations.

### Study site and sampling

**Figure 1:**
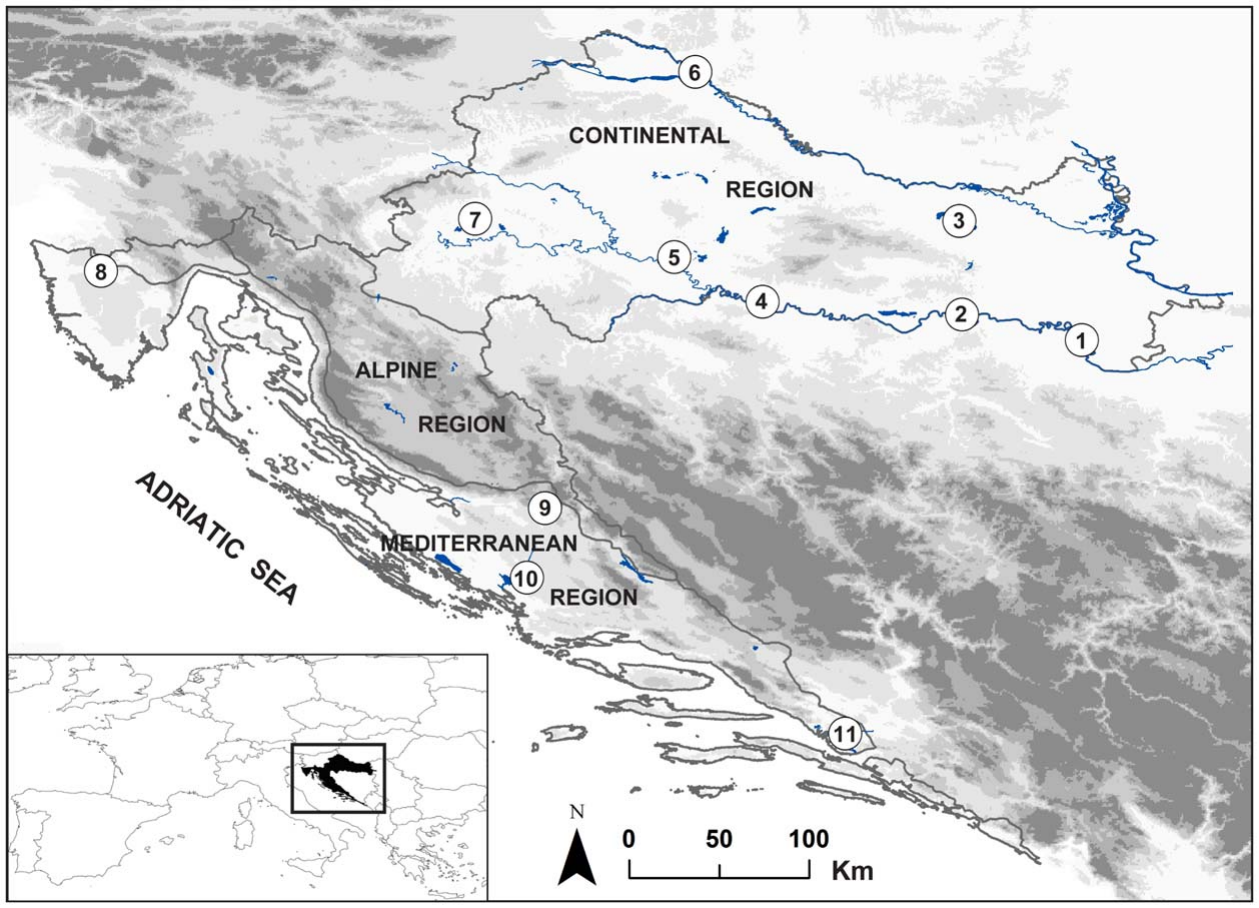
Map of the study area with location of the sampled *Fraxinus angustifolia* populations in Croatia. See Table 1 for population codes. The indicated boundaries of the biogeographical regions are based on the European Environment Agency (http://www.eea.europa.eu) and were adapted by the Croatian SINP (http://www.dzzp.hr/eng).

This study was conducted across the entire species' natural distribution range in Croatia (≈56.538 km^2^ land surface). Sampling was carried out in 11 natural populations of *F. angustifolia* (Figure 1), with about 30 individuals sampled per population (total n = 345) (Table 1). To avoid the sampling of close relatives, the minimum distance between sampled individuals was at least 50 m. Coordinates were recorded for each sampled tree using GPS. Trees were sampled from seven Continental and four Mediterranean natural stands, comprising the whole environmental gradient in Croatia in which the species occurs (Figure 1). All populations from the Mediterranean region where stands had sufficient size to allow at least 30 individuals to be sampled were included.

**Table 1:** Sampling sites and statistics of genetic variation for 11 *Fraxinus angustifolia* populations in Croatia at six microsatellite loci.

Population No.	Biogeographical Region	Population name	Lat (DD)	Long (DD)	n	*N*_a_	*N*_ar_	*N*_pr_	*H*_O_	*H*_E_	*H*_Enull_
1	Continental	Županja	18.742	45.003	30	13.00	12.67	1	0.650	0.672	0.710
2	Continental	Trnjani	18.144	45.131	29	12.67	12.56	4	0.733	0.724	0.726
3	Continental	Durđenovac	18.130	45.598	32	15.33	14.42	8	0.708	0.748	0.777
4	Continental	Stara Gradiška	17.147	45.196	31	13.33	12.81	5	0.697	0.682	0.686
5	Continental	Lonjsko polje	16.706	45.418	32	13.83	13.24	3	0.696	0.720	0.750
6	Continental	Čakovec	16.810	46.343	31	17.83	17.15	7	0.737	0.771	0.772
7	Continental	Jastrebarsko	15.711	45.611	32	15.17	14.44	2	0.754	0.715	0.727
8	Mediterranean	Mirna	13.840	45.349	32	13.33	12.67	0	0.656	0.681	0.684
9	Mediterranean	Zrmanja	16.059	44.167	32	10.67	10.20	3	0.625	0.668	0.688
10	Mediterranean	Krka	15.969	43.819	32	12.83	12.23	3	0.682	0.693	0.695
11	Mediterranean	Neretva	17.559	43.040	32	10.17	9.81	1	0.578	0.621	0.642
1–7		Continental			217	14.45	13.90	57	0.711	0.720	0.735
8–11		Mediterranean			128	11.75	11.23	9	0.635	0.666	0.677
		P†					0.03		0.02	0.03	
1–11		Overall mean				13.47	12.93	3.36	0.683	0.699	0.714

n - sample size; *N*_a_ - average number of alleles per locus; *N*_ar_ - allelic richness; *N*_pr_ - total number of private alleles; *H*_O_ - observed heterozygosity; *H*_E_ - expected heterozygosity; *H*_Enull_ - expected heterozygosity calculated on allele frequencies corrected for null-alleles;

† P-value of the permutation tests for differences between the putative ecotypes for *N*_ar_, *H*_O_, and *H*_E_.

About 9% of Croatian land area is frequently flooded [35], and natural floodplains of the Continental region represent the main habitat for the species. Floodplain forests in Croatia, dominated by narrow-leaved ash or mixed stands with oaks, are one of the most preserved in Europe. The majority of these forests stretch along the Sava, Kupa, Drava, and Danube rivers, all belonging to the Black Sea catchment (35.133 km^2^). In this region, stands are influenced by hot summers and cold winters with medium precipitation levels. In contrast, coastal stands are small and fragmented, influenced by the Mediterranean climate which is characterised by hot, dry summers and mild, rainy winters. These forests grow along the rivers of the Adriatic catchment area (21.405 km^2^), the Dinaric karst fields and wetland sites (Mirna, Krka, Zrmanja, Cetina, and Neretva).

### Microsatellite analysis

Total DNA was extracted using DNeasy 96 Plant Kit (Qiagen) from up to ten mg of dried leaf tissue following the manufacturer's protocols. We used six polymorphic microsatellite markers that had been extensively used in *Fraxinus* spp. studies: Femsatl4, Femsatl11, Femsatl12, Femsatl16, Femsatl19 and M2–30 [36], [37]. For Femsatl12, the redefined primers of Gerard [24] were used to avoid null alleles seen with the original primer set. The PCR conditions followed those used by Morand [22]. Fluorescent labelling of the forward primers enabled the detection of PCR products by capillary electrophoresis (ABI 3100), and allele sizes were scored using GeneMapper 4.0 (Applied Biosystems).

### Population genetic diversity and structure

GENEPOP 4.0 [38] was used to estimate the following genetic diversity parameters: average number of alleles per locus (*N*_a_), observed heterozygosity (*H*_O_), and expected heterozygosity (*H*_E_). The program MICRO-CHECKER [39] was used to check for potential problems related to allele dropout and the presence of null alleles. The estimates of the null allele frequencies were based on the expectation-maximisation algorithm [40] and then calculated using FREENA [41]. The adjusted allele frequencies were used to recalculate the expected heterozygosity values (*H*_Enull_). GENEPOP was also used to test for Hardy-Weinberg equilibrium (HWE) for each locus in each population and to test the loci for linkage disequilibrium. The probability tests were based on the Markov chain method [42]. The sequential Bonferroni adjustment [43] was applied to correct for the effect of multiple tests using SAS (SAS Ver. 9.1; SAS Institute Inc., Cary, NC, USA). FSTAT Ver. 2.9.3.2 [44] was used to calculate allelic richness (*N*_ar_), which yields allele counts standardised to the minimum sample size, and to test the significance of the differences in average values of *N*_ar_, *H*_O_ and *H*_E_ between the Continental and Mediterranean populations. The number of private alleles (*N*_pr_) per population was assessed by MICROSAT [45].

Pairwise genetic distances between populations (*F*_ST_) and their significance were calculated using FSTAT. Pairwise *F*_ST_ values were also estimated after correcting for the presence of null alleles using a method implemented in FREENA [41]. The overall population genetic structure was estimated for each locus and as a multilocus estimate with Wright's *F*-statistics using Weir and Cockerham's method [46] implemented in FSTAT. The analysis of molecular variance (hierarchical AMOVA) was performed to examine the partition of microsatellite variation between the Continental and the Mediterranean regions, among populations within the regions, and within populations using Arlequin Ver. 3.5.1.2 [47]. The variance components were tested by non-parametric randomisation tests using 10 000 permutations.

### Association between genetic variation and environmental heterogeneity

#### Species presence data

We obtained 335 occurrence points from 11 sampled populations. Further locality records were obtained from the Flora Croatica Database (FCD, http://hirc.botanic.hr/fcd/, n = 60), the National Forest Inventory (n = 352, [48]), and personal communications (n = 55, see acknowledgements). In total, we compiled 802 high resolution species occurrence points.

#### Environmental data

Climate data for current conditions were obtained from the WorldClim database with a spatial resolution close to a square km [49]. First, the correlations among all 19 WorldClim bioclimatic variables and topographic variables for all presence points were calculated to exclude the highly correlated ones (*r*>0.75), whilst keeping the variables useful in predicting the distribution limits of trees, such as climatic averages and extremes [50].

Ten environmental variables were selected to describe the ecological characteristics of the sampled stands, for the Principal Component Analysis (PCA) and for the calculation of environmental distances. The eight bioclimatic variables included averages, extremes and seasonal variation in precipitation and temperature, and the two topographic variables altitude and terrain slope (see Table 2). Three additional layers were included in the construction of the ENMs as predictors: terrain aspect, a distance-to-water variable, and habitat type (see Table 3). Because of its circular nature, terrain aspect was recalculated and presented as two variables, northness and eastness [51]. Rasterised layers of distance-to-water and habitat types were obtained from the Croatian Wetlands and Habitat Map GIS databases maintained by the State Institute for Nature Protection (SINP; http://www.cro-nen.hr/map/index_en). All topographic variables were based on a 90-m spatial resolution digital elevation model (DEM) (Shuttle Radar Topography Mission; http://www2.jpl.nasa.gov/srtm) and prepared in ArcGIS® 9.3 (ESRI).

**Table 2:** Pearson correlation coefficients between ten environmental variables and scores of the first three principal components.

Environmental variables		Principal component					
		PC1		PC2		PC3	
BIO 01	Annual Mean Temperature	−0.978	***	−0.106	**	−0.047	^ns^
BIO 04	Temperature Seasonality (standard deviation*100)	0.896	***	−0.117	**	−0.288	***
BIO 05	Max Temperature of Warmest Month	−0.849	***	−0.162	***	−0.363	***
BIO 06	Min Temperature of Coldest Month	−0.954	***	−0.051	^ns^	0.063	^ns^
BIO 12	Annual Precipitation	−0.706	***	0.115	**	0.665	***
BIO 15	Precipitation Seasonality (Coefficient of Variation)	−0.779	***	0.254	***	−0.232	***
BIO 18	Precipitation of Warmest Quarter	0.744	***	−0.108	**	0.595	***
BIO 19	Precipitation of Coldest Quarter	−0.929	***	0.131	***	0.248	***
DEM 30	Digital elevation model	0.351	***	0.765	***	0.143	***
DEM S 30	Slope	0.036	^ns^	0.724	***	−0.263	***
	Eigenvalue	6.03		1.27		1.22	
	% of variance	60.32		12.70		12.23	

“***” significance at the 0.1% nominal level,

“**” significance at the 1% nominal level,

“*” significance at the 5% nominal level, “ns” non-significant values.

**Table 3:** Environmental variables used for *Fraxinus angustifolia* ENMs based on the Maximum Entropy (Maxent) method.

		Variable contributions (%)			
Environmental variables		Overall ENM	Continental ENM	Mediterranean ENM	Source
BIO 1	Annual Mean Temperature (°C)	1.1	1.2	38.0	www.worldclim.org
BIO 4	Temperature Seasonality (standard deviation *100)	1.0	12.7	4.1	www.worldclim.org
BIO 5	Max Temperature of Warmest Month (°C)	11.2	1.1	1.1	www.worldclim.org
BIO 6	Min Temperature of Coldest Month (°C)	0.3	0.1	0.6	www.worldclim.org
BIO 12	Annual Precipitation (mm)	2.9	0.8	0.1	www.worldclim.org
BIO 15	Precipitation Seasonality (Coefficient of Variation)	1.6	0.9	0.2	www.worldclim.org
BIO 18	Precipitation of Warmest Quarter (mm)	2.6	5.0	2.6	www.worldclim.org
BIO 19	Precipitation of Coldest Quarter (mm)	1.4	8.1	0.2	www.worldclim.org
DEM	Digital elevation model (elevation in m)	15.2	26.7	1.6	www2.jpl.nasa.gov/srtm
DEM S	Slope (degrees)	0.3	0.3	0.4	generated from DEM
DEM_ae	aspect eastness (eastness index)	0.5	0.2	0.1	generated from DEM
DEM_an	aspect northness (northness index)	0.5	0.3	0.1	generated from DEM
NKS	habitat type (as categorical variable)	21.2	26.9	1.6	www.cro-nen.hr/map
Dwater	Distance to water (m)	40.2	16.0	49.3	www.cro-nen.hr/map

Relative contributions of environmental variables to each of the three ENMs are shown as averages over ten replicate runs.

#### Correlation between genetic, geographic and environmental distances

To generate the environmental distance matrix, we performed a canonical discriminant analysis (CDA) based on the ten environmental variables (see Table 2) using PROC CANDISC in SAS. Squared Mahalanobis distances (D^2^) between the populations were computed to obtain a matrix of environmental distances among the populations. Mahalanobis distances are analogous to Euclidian distances but also account for covariance among variables. Mantel tests [52] were used to examine the extent to which the neutral genetic structure can be explained by the environmental heterogeneity. We computed and tested the correlations between (1) the matrix of the natural logarithm of geographical distances (in km) between pairs of populations and the matrix of pairwise *F*_ST_/(1-*F*_ST_) ratios and (2) the matrix of environmental distances (D^2^) and the matrix of pairwise *F*_ST_/(1-*F*_ST_) ratios. Only individuals that had information about all three parameters (genetic, geographic and environmental) were used for the correlation tests (*n* = 335). In addition, a three-way Mantel test was applied between the matrix of environmental distances and the matrix of pairwise *F*_ST_/(1-*F*_ST_) ratios while accounting for geographical distances among populations. The significance level was assessed after 10,000 permutations as implemented in NTSYS-pc Ver. 2.02 [53].

Finally, the relationships between the populations based on both genetic distances and environmental distances (D^2^) were visualised by constructing two neighbour-joining trees. Pairwise Nei's standard genetic distances [54] were calculated and an unrooted phylogenetic tree was constructed using the Neighbour-joining algorithm with 1 000 bootstrap replicates over microsatellite loci as implemented in the software PHYLIP Ver. 3.6b [55].

### Ecological niche analyses

The total set of 802 occurrence points was used to further examine the levels of ecological niche divergence between the populations from the Continental and Mediterranean biogeographical regions. Each occurrence point was assigned to a specific region as shown in Figure 1. First, we conducted the PCA based on ten environmental variables (Table 2) that describe the ecology of all presence localities using PROC PRINCOMP in SAS. Second, we generated an overall ENM based on all 802 presence points. In addition to the ten variables used for the PCA, terrain aspect, distance-to-water, and categorical habitat type variable were added (Table 3). All environmental layers were resampled to be used at a 100 m×100 m spatial resolution.

We applied a maximum entropy presence-only modelling technique to estimate the ecological niche of the species using Maxent Ver. 3.3.2 [56], [57]. This method has proven robust for presence-only data [58]. We performed ten replicate runs using cross-validation with default parameters and we used a logistic output from Maxent [57]. Model performance was evaluated using the area under the curve (AUC) of a receiver-operating characteristic (ROC) plot. To depict suitable habitat maps, we used the minimum training presence threshold [56]. Finally, the relative contributions of the environmental variables to the Maxent model were recorded.

To further assess whether populations from different regions occupy divergent ecological niche space, we simulated two separated ENMs using only occurrence data from either the Continental or Mediterranean region following the same modelling procedures. To determine whether any areas of overlap between the putative ecotypes exist, we summed the probabilities of occurrence from the two regional ENMs after applying the minimum training presence threshold to each. In this way, we evaluated whether the stands present in the predicted overlap zones were congruent with low values of pairwise genetic distances. All resulting models were visualised in ArcGIS® 9.3.

## Results

### Within population genetic diversity

A total of 176 alleles were observed across the six markers, with the number of alleles per locus ranging from nine (Femsatl16) to 48 (M2–30) and a mean value of 29.33. *N*_ar_ ranged from 9.81 to 17.15 (Table 1). High levels of both within populations *H*_O_ and *H*_E_ were found (mean values over loci and populations were 0.683 and 0.699, respectively). There was no evidence of allele dropout in the data according to MICRO-CHECKER. Null alleles were suggested in seven out of 66 locus×population combinations. Estimated null allele frequencies using FREENA ranged from 0.052 (Femsatl12 in population Trnjani) to 0.146 (Femsatl16 in population Lonjsko Polje). The *H*_E_ values increased slightly when recalculated using adjusted allele frequencies (Table 1), but no significant differences were observed between values of *H*_E_ and *H*_Enull_ in any of the analysed populations (Kruskal-Wallis test, *P* = 0.52–0.81). Therefore, all subsequent analyses were conducted using the original data set. Significant differences (*P*<0.05) in genetic diversity (mean *N*_ar_, *H*_O_ and *H*_E_) were found between the Mediterranean and Continental populations, with lower values observed in the Mediterranean stands. Moreover, 57 private alleles were identified in the Continental region, whereas there were only nine in the Mediterranean region (Table 1). No significant departures (*P*<0.01) from the HWE were observed at any loci in any population after applying sequential Bonferroni corrections. Finally, among a total of 165 tests for linkage disequilibrium between pairs of loci, no test was found significant after applying sequential Bonferroni corrections (*P*<0.01).

### Genetic structure among populations and biogeographical regions

Although testing for HWE within each population showed no significant departures, the average multilocus inbreeding coefficient of the overall sample was slightly positive but significant (*F*_IS_ = 0.024, *P* = 0.0025). Moreover, mean multilocus values of *F*_IS_ in the Mediterranean region were almost four times higher and significant than that for the Continental region (Table 4). The overall multilocus differentiation among all populations (*F*_ST_) was 0.022. The within-region *F*_ST_, however, was higher in the Mediterranean populations (*F*_ST_ = 0.027) than that among the Continental populations (*F*_ST_ = 0.012), showing that the coastal populations were more structured.

**Table 4:** *F*-statistics among 11 *Fraxinus angustifolia* populations and within each of the biogeographical regions.

Locus	*F*_IT_	*F*_IS_	*F*_ST_
FEM11	0.028	0.011	0.017
FEM16	0.222	0.212	0.013
FEM19	−0.004	−0.053	0.047
FEM4	−0.082	−0.094	0.011
FEM12	0.165	0.145	0.024
M230	0.033	0.011	0.021
Multilocus estimates	**0.045**	**0.024**	**0.022**
Permutation test	P<0.0001	P = 0.0025	P<0.0001
Continental region			
Multilocus estimates	0.024	0.012	**0.012**
Permutation test	P = 0.0104	P = 0.1287	P<0.0001
Mediterranean region			
Multilocus estimates	**0.071**	**0.046**	**0.027**
Permutation test	P<0.0001	P = 0.0024	P<0.0001

*F*_IT_ - overall inbreeding coefficient; *F*_IS_ - average inbreeding coefficient; *F*_ST_ - differentiation among populations. Significant values are indicated in bold.

Pairwise *F*_ST_ values ranged from zero between Trnjani/Županja to 0.074 between Čakovec/Neretva population pairs (Table 5). No significant differences were observed between raw pairwise *F*_ST_ and pairwise *F*_ST_ corrected for null alleles (Kruskal-Wallis test, *P* = 0.79), suggesting that null alleles did not affect this analysis. Most population pairs from the Continental region had non-significant pairwise *F*_ST_ values, with the exception of the Čakovec population. In contrast, most population pairs within the Mediterranean were significantly differentiated. Pairwise differentiation was also detected between the Mediterranean and Continental populations, with the exception of the Istrian population Mirna, which was not significantly differentiated from several Continental populations. The AMOVA analysis (Table 6) showed that most of the genetic diversity was attributable to differences among individuals within populations (97.36%). However, a small but highly significant percentage of variation was explained by differences among populations within regions (1.72%) and by differences between regions (0.92%), confirming the geographic structuring of populations.

**Table 5:** Pairwise *F*_ST_ values (lower diagonal) and null-allele corrected *F*_ST_ values (upper diagonal) among 11 *Fraxinus angustifolia* populations.

No	Population name	1	2	3	4	5	6	7	8	9	10	11
1	Županja		0.001	0.006	0.010	0.013	0.025	0.004	0.010	0.027	0.011	0.036
2	Trnjani	0.000^ns^		0.004	0.011	0.009	0.020	0.002	0.008	0.033	0.016	0.038
3	Durđenovac	0.007^ns^	0.002^ns^		0.009	0.014	0.023	0.003	0.014	0.022	0.019	0.032
4	Stara Gradiška	0.010^ns^	0.011^ns^	0.005^ns^		0.018	0.029	0.010	0.015	0.032	0.020	0.035
5	Lonjsko polje	0.013^ns^	0.006^ns^	0.014^ns^	0.014*		0.029	0.004	0.025	0.038	0.035	0.045
6	Čakovec	0.027**	0.020**	0.021**	0.028**	0.026**		0.028	0.032	0.050	0.031	0.076
7	Jastrebarsko	0.006^ns^	0.001^ns^	0.003^ns^	0.009^ns^	0.003^ns^	0.027**		0.008	0.027	0.018	0.026
8	Mirna	0.009^ns^	0.008^ns^	0.011^ns^	0.014*	0.020*	0.031**	0.007^ns^		0.024	0.015	0.030
9	Zrmanja	0.029**	0.032**	0.023**	0.030**	0.039**	0.049**	0.028**	0.022**		0.028	0.035
10	Krka	0.011**	0.016**	0.018**	0.020**	0.032**	0.031**	0.018**	0.016**	0.027**		0.031
11	Neretva	0.038**	0.037**	0.032**	0.033**	0.045**	0.074**	0.028**	0.027**	0.038**	0.032**	

*P*-values as obtained by randomisations:

“**” significance at the 1% nominal level,

“*” significance at the 5% nominal level, “ns” non-significant values.

**Table 6:** Analysis of molecular variance (AMOVA) for the partitioning of genetic diversity among and within populations of *Fraxinus angustifolia* grouped into two biogeographical regions (Continental vs. Mediterranean).

Source of variation	df	Variance components	Percentage of variation	φ-statistics	P(φ)
Among regions	1	0.020	0.92	φ_CT_ = 0.009	<0.0001
Among populations within regions	9	0.037	1.72	φ_SC_ = 0.017	<0.0001
Within populations	679	2.086	97.36	φ_ST_ = 0.026	<0.0001

P(φ) - φ-statistics probability level after 10 000 permutations.

### Association between genetic and environmental variation

**Figure 2:**
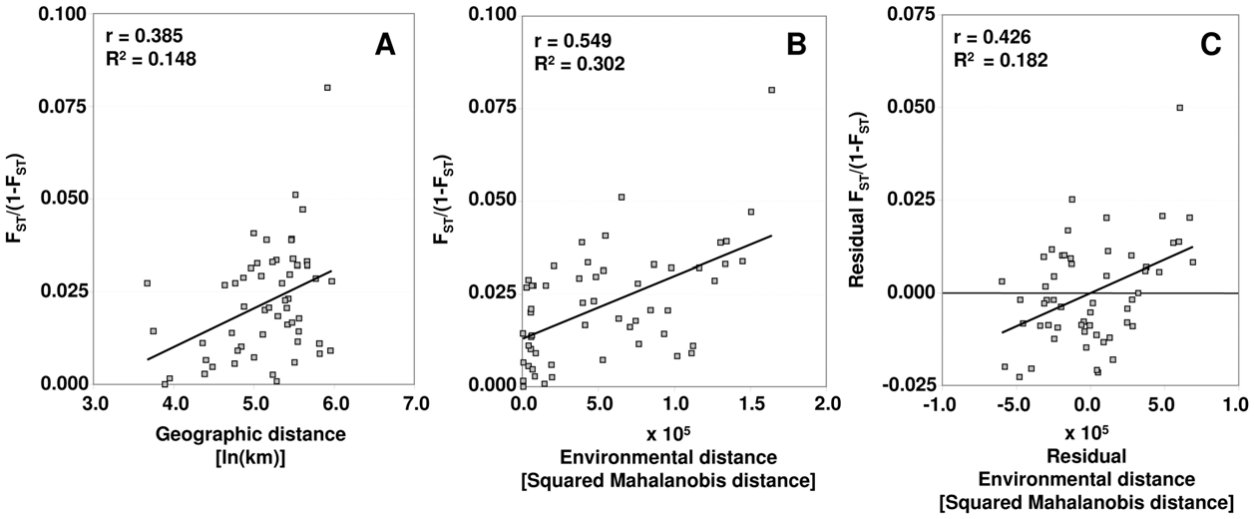
Isolation-by-distance and Isolation by environmental distance. Plots of simple and partial Mantel tests showing the relationships between A) geographic and genetic distances, B) environmental and genetic distances, and C) residual environmental and genetic distances, by taking into account the geographic distances among 11 populations of *Fraxinus angustifolia*.

The analysed populations showed both significant levels of IBD (*r* = 0.385, *P* = 0.026) (Figure 2A) and even higher correlation between genetic divergence and environmental distance (*r* = 0.549, *P* = 0.004) (Figure 2B). The correlation between genetic and environmental distances remained significant (*r* = 0.426, *P* = 0.002) even after accounting for the effect of geographical distance in a three-way Mantel test (Figure 2C). On the other hand, the removal of the effect of environmental variation in the partial Mantel test resulted in a non-significant correlation between genetic and geographic distances (*r* = −0.025, *P* = 0.438). Therefore, our populations show a clear “isolation by environmental distance” pattern, rather than IBD as such.

### Ecological niche analyses

#### PCA analyses

**Figure 3:**
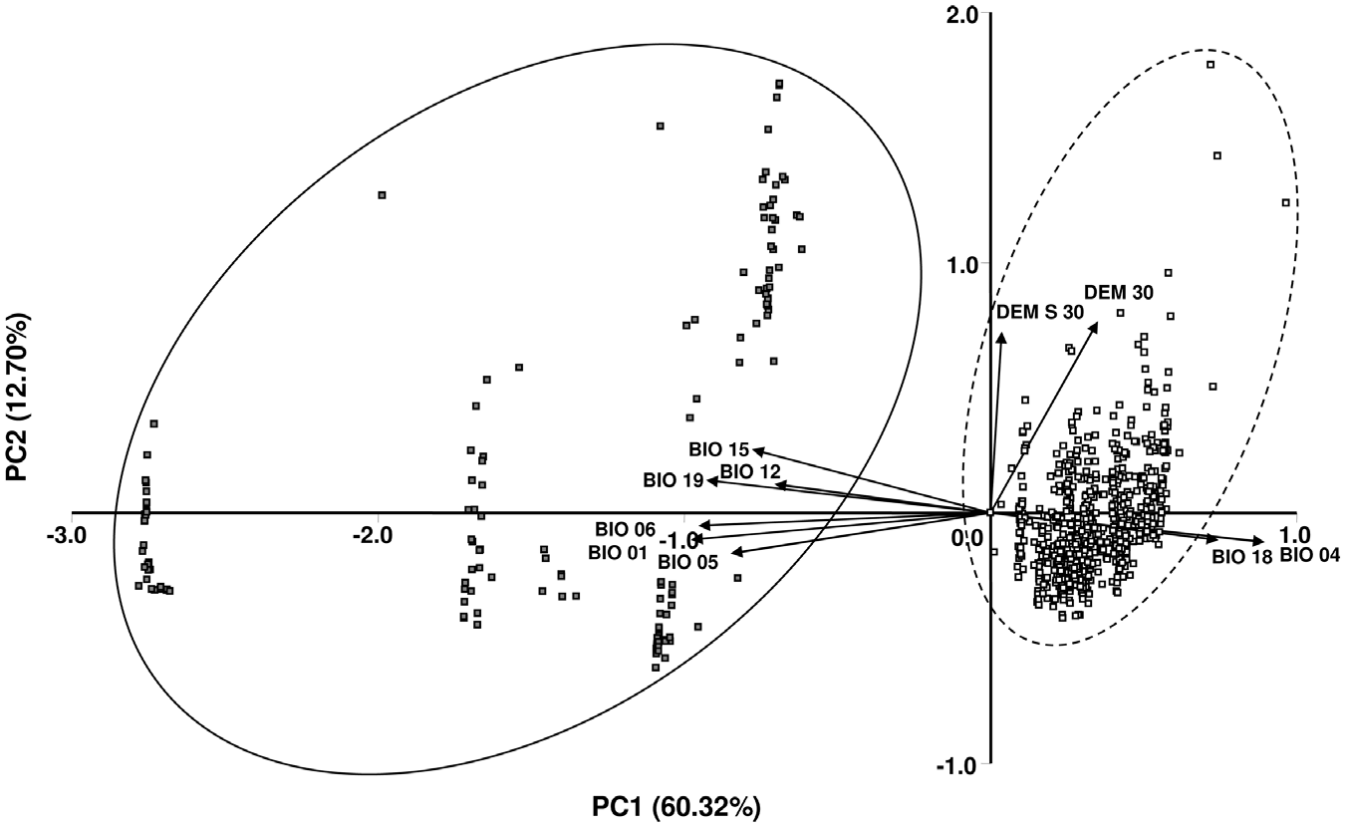
Plot of PCA based on ten environmental variables describing 802 *Fraxinus angustifolia* localities. The Continental (white squares) and Mediterranean (grey squares) ecological lineages were separated along the PC1 and PC2. See Table 2 for environmental variable codes.

The first principal component (PC1) explained 60.32% of the total variation and clearly separated the Continental and Mediterranean localities along temperature and precipitation gradients (Figure 3). Elevation and slope were highly positively correlated with PC2, explaining 12.70% of the total variation and reflecting a topographic gradient. In addition, PCA on environmental data revealed a notable environmental sub-structure within the Mediterranean region, where each of the populations clustered along a different river valley. The results show that Continental populations occupy habitats that are cooler with lower winter temperatures (below zero) and higher summer rainfall. In contrast, coastal populations are characterised by warmer habitats with higher winter temperatures and wetter winters. Generally, the range of variation in the examined environmental variables appears to be more pronounced between the Mediterranean localities than among the Continental localities.

#### Ecological niche modelling

**Figure 4:**
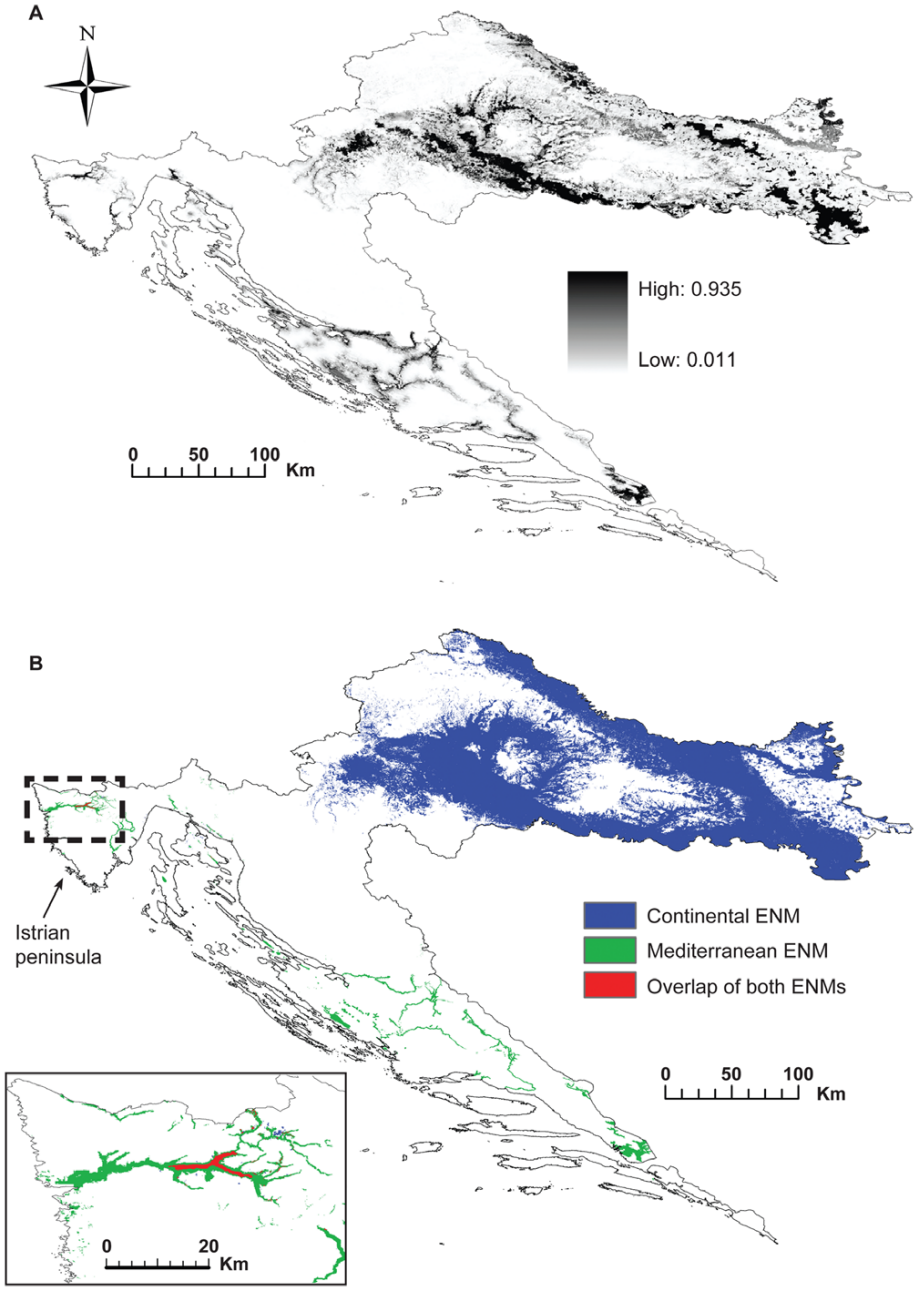
Predicted Maxent Ecological niche models (ENMs) for *Fraxinus angustifolia*. A) Overall ENM. Colour levels of shading from white (unsuitable habitat) to black (highest suitability) represent the continuous species' probability distribution after thresholding. B) Overlay of the two independently predicted regional ENMs to identify areas of environmental overlap (highlighted by the enlarged box). Minimum training presence thresholds: Continental = 0.012, Mediterranean = 0.044.

The Maxent model performed well with high average training and test AUC values across ten replicate runs (Table 7) and was congruent with the currently known distribution of *F. angustifolia* in Croatia (Figure 4A). The highest probabilities of occurrence were in the Continental region, in lowlands along large rivers (Sava and Drava) with more or less continuous distribution. In contrast, the predicted distribution was discontinuous in the Mediterranean region with several isolated areas of high suitability associated with shorter karst river valleys along the eastern Adriatic coast (Mirna, Krka, Zrmanja, and Neretva). The overall model did not predict suitable habitats in the Alpine region, confirming that that the species distribution is strongly associated with river valleys and wetland sites (Table 3).

**Table 7:** Evaluation of each ENM using a threshold-independent ROC analysis with AUC.

	N presence points	Average training values	Average test values
		AUC; SD	AUC; SD
Overall ENM	802	0.948; 0.007	0.942; 0.008
Continental ENM	144	0.956; 0.006	0.951; 0.004
Mediterranean ENM	658	0.993; 0.008	0.985; 0.019

SD is the standard deviation of the average AUC values after ten replicated Maxent runs.

The overlap between the two regional modelled distributions was very low (Fig. 4B), suggesting strong regional niche differentiation between the two putative ecotypes. Despite high AUC values, each model alone predicted a highly reduced distribution of the species in comparison with the overall model. Contrary to our expectations, the variables differed in their contributions to the three distribution models (Table 3). Distance-to-water contributed most to the overall and Mediterranean ENM, whereas habitat type and elevation were most important predictors for the Continental ENM.

#### Neighbour-joining analysis

**Figure 5:**
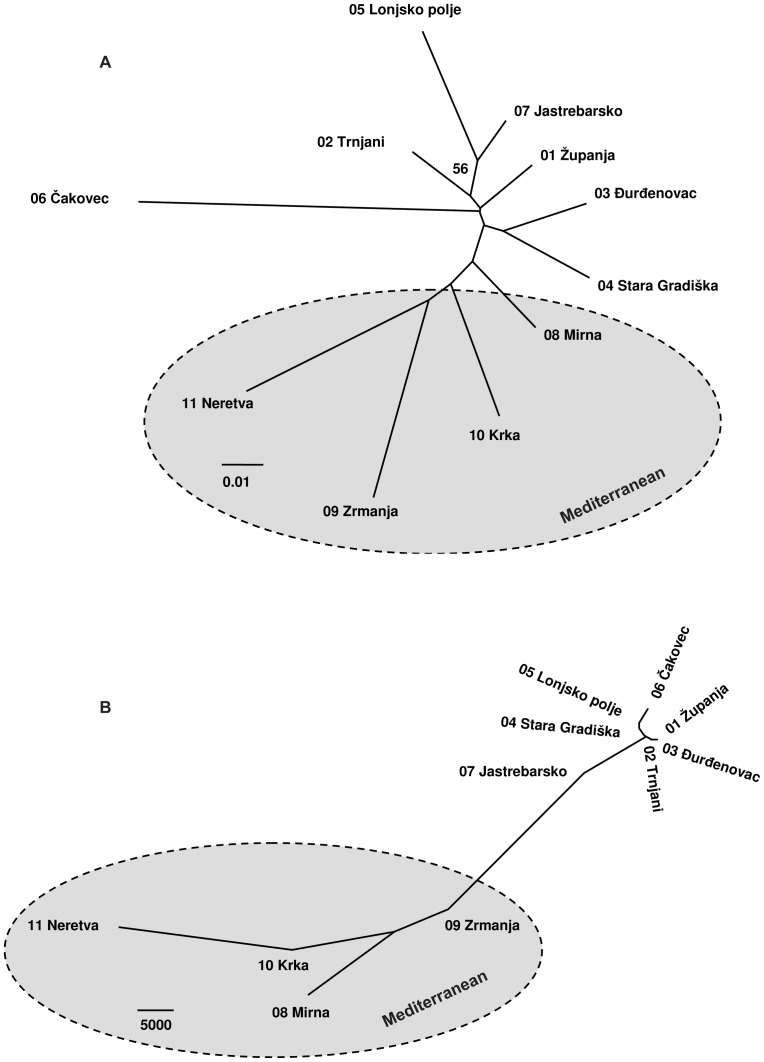
Unrooted neighbour-joining trees for *Fraxinus angustifolia* populations. A) Based on Nei's genetic distance. B) Based on environmental distance.

Trees based on either genetic distances (Figure 5A) or environmental distances (Figure 5B) were congruent in their major features, suggesting that environmental variation may promote genetic divergence of the studied populations. Moreover, a comparison of the genetic tree (Figure 5A) with the overlap map of the regional ENMs (Figure 4B) showed that the coastal population Mirna, which is situated intermediately in the genetic tree, is located in the overlap area. There are, however, some incongruencies. For example, the Jastrebarsko population belongs to the Continental region based on genetic markers but can be considered intermediate from an ecological point of view.

## Discussion

Our combined analysis of genetic data, multivariate statistics on environmental data and ENM suggests that current genetic variation patterns in natural *Fraxinus angustifolia* populations in Croatia may be influenced by the local ecological conditions rather than by geographic distances only. We observed an overall pattern of significantly higher genetic diversity in the Continental region and low local differentiation that contrasts with the reduced genetic diversity and stronger structuring in the Mediterranean region. The extent of potential ecological niche overlap between the continental and coastal populations was low, suggesting that two ecologically distinct lineages of narrow-leaved ash may occur in the study area. ENM was in agreement with the genetic distance analysis, which found that most Continental and Mediterranean populations were differentiated.

### Genetic diversity and structure

The genetic analysis results agree with the expectations of high polymorphism within populations and low genetic differentiation between populations, as observed in ashes and wind-pollinated trees in general [21], [26], [59], [60]. However, the large and significant local homozygote excess (*F*_IS_>0.15) found in many European *F. excelsior* populations (22, 26, 59) was not observed in the present study. This characteristic of common ash was often attributed to the presence of null alleles, biparental inbreeding or the Wahlund effect. Unlike common ash populations studied in Europe [21], [22], [26], [59] all of the populations studied herein were in HWE, indicating weak evidence for the presence of null alleles or of local inbreeding. Finally, the average multilocus inbreeding coefficient *F*_IS_, which provides information on the cumulative effect of inbreeding, was low but significant at the level of the overall sample (average *F*_IS_ of 0.024), but at the regional level only for the Mediterranean stands (average *F*_IS_ of 0.046). Although positive *F*_IS_ values may reflect the presence of null alleles, which are commonly suspected for microsatellite loci, controlled crosses carried out by Morand [22] showed that most of the loci used in this study do not show null alleles. Moreover, there were no significant differences between the *H*_E_ and *F*_ST_ values calculated with and without corrections for null allele frequencies. A plausible explanation for this positive inbreeding would be a Wahlund effect at the level of the overall sample population, mainly due to the differentiation between subpopulations, even though subpopulations themselves are in HWE.

Local population sizes determined by suitable habitat availability may explain the contrasting genetic structures found within and among biogeographical regions. The observed higher genetic diversity in the Continental populations can possibly be maintained due to larger effective population sizes compared to their Mediterranean counterparts. Longitudinal distribution along rivers and floodplains typical for the Pannonian lowlands with no barriers to gene flow allows free pollen and seed dispersion between the populations, which explains the lack of significant genetic structure in this region based on pairwise *F*_ST_, even between most distant populations. On the other hand, Mediterranean populations are reduced to a few smaller suitable sites associated with Dinaric karst fields, short karst rivers and rare natural wetlands with no apparent above-ground connections due to the limestone base. In consequence, populations are more isolated from each other and also from the Continental part of the distribution, limiting dispersion and favouring the maintenance of an intra-regional genetic structure.

### Genetic divergence and environmental heterogeneity

Large amounts of neutral population genetic variation were explained by environmental variation rather than by simple geographic distances, suggesting a strong role of environmental heterogeneity in the genetic divergence of populations [6]–[9], [61]. First, pairwise *F*_ST_ values and multilocus estimates of *F*-statistics suggest that each region has different evolutionary constraints. For example, some very close Mediterranean populations (such as Zrmanja and Krka) have significant pairwise *F*_ST_ values although they are not geographically distant (only 40 km apart), suggesting more restricted gene flow in this region probably caused by isolation of suitable habitat and/or habitat differentiation. Environmental distinctiveness and habitat discontinuities among Mediterranean populations are apparent from the PCA and niche models, revealing a similar differentiation pattern compared to pairwise *F*_ST_. The Mediterranean is known for pronounced environmental heterogeneity over very short distances because of factors such as slope, exposure, distance from sea, and rock type. In contrast, most of the Continental populations that occur in a rather homogenous environment exhibit non-significant pairwise *F*_ST_ values (except for the Čakovec population), even in distant populations (>240 km). Similar trends were observed in *Taxus baccata* [62] on a broader geographical scale, in which populations located in the stronger Mediterranean climate displayed higher pairwise differentiation within regions than those from the inland areas, suggesting that the geographical and environmental features can influence population divergence of different tree species in this area.

Second, we found a significant correlation between genetic and environmental variation. IBED patterns may be arising from a neutral process of temporally disrupted gene flow among individuals living in environmentally distinct habitats, leading to phenological differences [7], [22]. Gene flow should homogenise neutral genetic variation in wind-pollinated tree species at short geographical distances, but habitat differentiation can act as a barrier to gene flow, causing environmental isolation and genetic differentiation of spatially close plant populations [5], as found in our study. Finally, a significant impact of genetic drift in these populations can be discounted as drift alone would create a random genetic structure that was not observed herein; instead, ecologically similar populations were also grouped genetically. In sum, we show that the observed genetic variation pattern is associated with environmental gradients. We recognize that the observed IBED pattern does not imply causality and this correlation might have other plausible or more complex interpretations which cannot be explicitly tested using our current data, like different population ages in two regions because of independent colonization events. Future tests with candidate genes for traits of interest could also clarify the possible role of natural selection in shaping the divergence of these populations, but such markers are only recently emerging for *Fraxinus* spp. [63]. Further exploration in our study species is currently underway.

### Ecological niche models and genetic structure

Using an ENM approach, we tested whether the predicted distributions of the species corresponded with the patterns of population genetic structure. In particular, we searched for areas where the Continental and Mediterranean ENMs overlap, as they could highlight populations from different biogeographical regions with lower levels of pairwise genetic differentiation. The overall ENM detected a separation of the two putative ecotypes by the mountainous region, representing an unsuitable habitat for the survival of the species and a potential barrier to gene flow. The Continental and Mediterranean ENM barely overlapped, indicating a clear divergence in the ecological niche space occupied by the populations in each region. An overlap of independent ENMs suggests only one point of contact in the Istrian peninsula at the Mirna population site (Figure 4B). This stand is indeed the only Mediterranean population with non-significant pairwise genetic distances towards most Continental populations and is located within an area of intermediate environmental conditions between the coast and the continent. Because one set of populations poorly predicts the distribution of the other set of populations and because their ecological niches almost do not overlap, our populations may represent two distinct evolutionary lineages, even with the low levels of genetic divergence [12], [16]. Lack of niche overlap in wide-ranging tree species may also appear to be driven by differences in abiotic conditions in different regions (soil, elevation, climate) [64]. Because our populations inhabit regions with clearly divergent climatic regimes and *F. angustifolia* has a wide distribution, both latitudinally and altitudinally, this is a confounding factor that needs to be kept in mind.

### Stability, migration crossroads and environmental heterogeneity in refugia

From our observations, we cannot exclude the fact that historical migration processes in this refugial area could have raised the observed pattern of genetic variation in studied populations. Mediterranean tree populations have persisted in the southern refugia without significant geographical movements due to long-term stable environmental conditions in the Mediterranean [65]. Hence, the lower genetic diversity, higher genetic differentiation and higher fixation rates observed in the Mediterranean populations could result from older populations with smaller historic population sizes persisting in environmentally more stable regions over longer periods [66]. Croatia and the wider area of the Dinaric Alps have already been identified as an important refugium for ash and other temperate tree species during the Pleistocene and stand on a contact zone of their different postglacial recolonisation routes [34], [67], [68]. Recent studies have also confirmed the existence of a ‘refugia within refugia’ pattern in these areas, where differentiation of distinct lineages on a small geographical scale has been observed (see [69] and references therein). Therefore, forests in this region are expected to harbour greater regional genetic diversity and uniqueness in comparison with the rest of their range [31], [65].

*F. angustifolia* is a thermophilic tree species with distinct moisture requirements that could have survived *in situ* during the last glacial period in several river valley sites along the Dalmatian coast at lower to mid-altitudes until today. These humid but not too cold sites provided continuous moisture availability and shelter for Mediterranean tree species during the Adriatic Sea level drop in the LGM, leaving the Northern half of the Adriatic Sea basin exposed and unsuitable for the survival of moisture-dependent species [33]. Northern coastal populations in Istria and the rest of the Continental populations could have been recolonised by expansion from the Dinaric Alps or from refugia in North Italy and/or Balkan Peninsula [68], most likely via the North Adriatic or along the Danube river lowlands. At least three *F. angustifolia* haplotype lineages meet at the vicinity of the investigated populations (H01, H02 and H03, *sensu* [68], [27], authors' personal observations), confirming that various migration events occurred in the past within the study area and suggesting that Croatian populations may have originated from various colonising routes that likely brought new diversity. Higher levels of genetic variation in the Continent could therefore be due to a gene flow among individuals from different glacial refugia in newly colonised regions [31] while southern coastal populations probably represent relict, genetically more divergent populations [65]. In fact, modelling of potential distribution of *Quercus robur* in Europe during the LGM [14] shows that both the Adriatic coastal and Continental lowland parts of Croatia were suitable for the survival of this ecologically similar species *in situ*. Assuming that *F. angustifolia* followed a similar distribution during the LGM, this species likely survived for long time in this area, allowing enough time for the differentiation of distinct populations to occur through a processes of ecological isolation.

## Conclusions

Overall, our results suggest that long-term stability of heterogeneous environments at regional spatial scales may explain current levels of genetic diversity and population genetic divergence in narrow-leaved ash in these ancient refugia. Environmental differences between the regions may have led to the general subdivision into two ecotypes, with the pronounced environmental heterogeneity in the Mediterranean further promoting the genetic differentiation of the coastal populations. Thus, the local genetic structure in the narrow-leaved ash is more complex than a simple allopatry divergence model as the populations are not clear-cut differentiated but rather in a complex genetic cline, probably resulting from the environmental heterogeneity over the studied geographical area.
